# Challenging Intervention to Restenosis of Right Coronary Ostial Stent Excessively Overhanging to the Aorta: A Case Report and Brief Review of Literature

**DOI:** 10.7759/cureus.25037

**Published:** 2022-05-16

**Authors:** Kerim Esenboğa, Ebru Şahin, Nil Özyüncü, Yakup Yamanturk, Sibel Turhan

**Affiliations:** 1 Cardiology, Ankara University School of Medicine, Ankara, TUR; 2 Cardiology, Bilecik Training and Research Hospital, Bilecik, TUR

**Keywords:** side strut, right coronary artery, double wire technique, acute coronary syndrome, aorto-ostial stent restenosis

## Abstract

Intervention of aorto-ostial stent restenosis is challenging due to coronary anatomical variations, possible non-selective ostial engagements, and difficulties crossing the central lumen. Aorto-ostial stent restenosis is intervened through the central lumen or protruding side struts using various techniques. These techniques are often difficult and complex such as double wire technique, balloon-assisted technique, snare technique, side-strut sequential ballooning technique, and guideline-facilitated side strut stenting technique. In this case report, we presented an intervention for right coronary artery (RCA) ostial stent restenosis using a combination of balloon-assisted double wire technique and side-strut stenting technique in an acute coronary syndrome patient.

## Introduction

Aorto-ostial lesions have high calcium and fibrous tissue content and have an increased elastic recoil [1]. Ostial anatomic variations, angiographic visualization of aorto-ostial origins, and difficulties in guiding catheter engagement lead to stenting of ostial lesions complex and challenging. Increased intimal hyperplasia or incomplete neointimalization and early and late stent malapposition are frequently observed after ostial lesion stenting. Precise stent positioning with negligible protrusion of stent struts into the aorta and adequate ostial coverage are shown to decrease late complications. Increased stent protrusion into the aorta results in challenging repeated interventions [2,3]. Extreme elastic composition, non-tubular anatomy, and increased elastic recoil of right coronary artery (RCA) ostium are the factors provocating in stent restenosis [1,4]. We present a challenging coronary intervention due to excessive stent overhung to the aorta in a patient with RCA ostial stent restenosis.

## Case presentation

**Figure 1:**
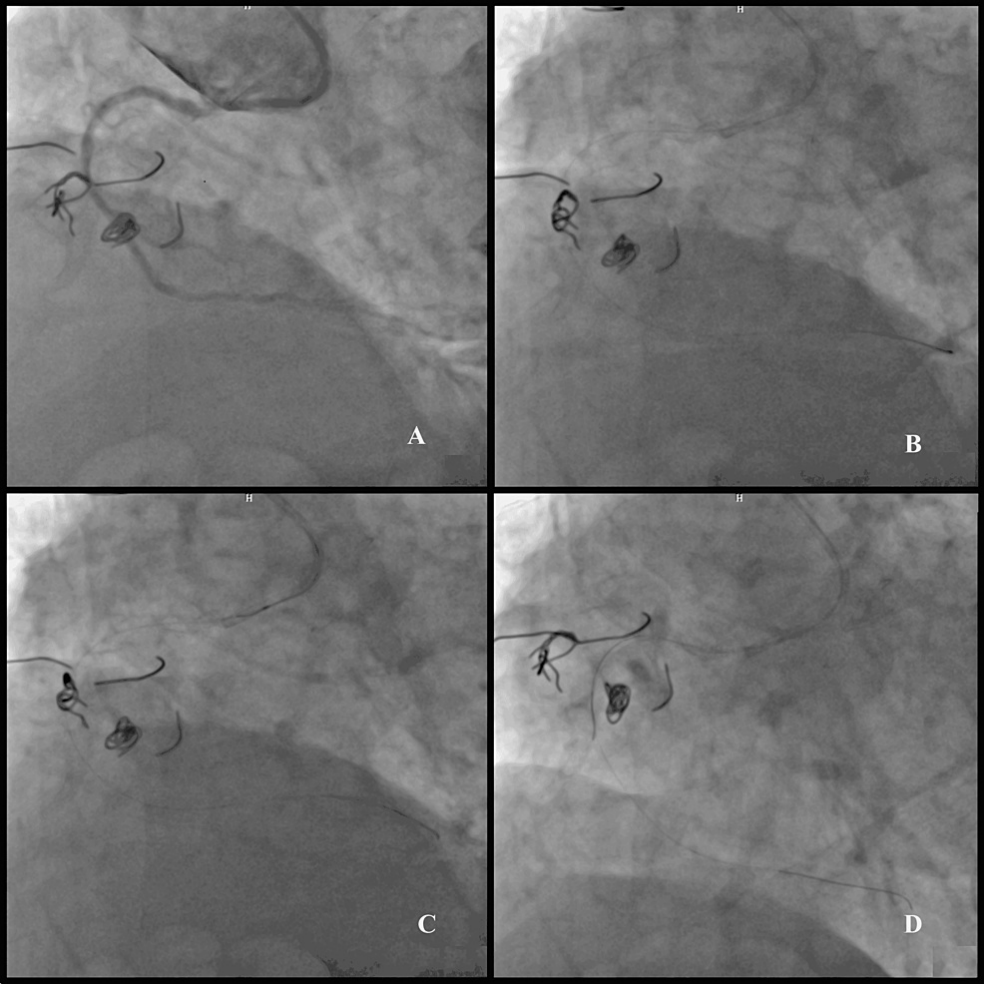
Right coronary artery (RCA) ostial lesion engagement and wiring (A) Non-selective engagement with 6 Fr AR 2 (Hialeah, FL: Cordis) guiding catheter; (B) distal side strut of the protruded part passed through with extra support guidewire; (C) double wire and balloon assisted technique used to cross the central lumen; (D) the second wire had passed through a more proximal side strut.

An 80-year-old male patient presented with progressive angina in form of pressure, which had been ongoing for five days and increasing frequency and severity. The patient had elevated troponin values and ST segment changes in ECG and was taken to the catheterization laboratory with the diagnosis of acute coronary syndrome (ACS). He had known coronary artery disease, with a history of percutaneous coronary intervention (PCI) to RCA six years ago. Angiography was planned for the patient. Before admission to the coronary angiography laboratory, informed consent was obtained for angiography consent and the use of images. Coronary angiography revealed non-critical atherosclerotic plaques at left anterior descending artery (LAD) and circumflex artery (Cx). Despite the use of many different catheters, the selective engagement of the RCA was not achieved. Non-selective injection of contrast into the aortic root showed the restenosis of RCA ostial stent and the proximal segment of the stent was excessively overhanging to the aorta. The closest non-selective engagement could be achieved with 6 Fr AR 2 (Hialeah, FL: Cordis) guiding catheter (Figure 1, panel A). The central lumen of the stent could not be passed through with the aid of extra support guidewire (Hi-Torque Whisper; Abbott Park, IL: Abbott Laboratories Inc.), but the distal side strut of the protruded part could be passed (Figure 1, panel B). Dilation of this side strut with 1.0 x 15 mm balloon over the wire was planned, but the balloon could not be advanced through the stent struts. This balloon was left on the first wire. We planned to take a second extra support guidewire (Hi-Torque Whisper) and cross the central lumen with the support of the first wire and with the aid of the balloon on the first wire (Figure 1, panel C).

The balloon was pushed through a little so that the catheter was retracted and brought to the coaxial angle with the ostium. By pushing the balloon catheter loaded on the first guidewire forward gently, guiding catheter was positioned to be more coaxial with the proximal edge of the protruded stent. However, it was observed that the second wire had passed through a more proximal side strut instead of the central lumen (Figure 1, panel D).

**Figure 2:**
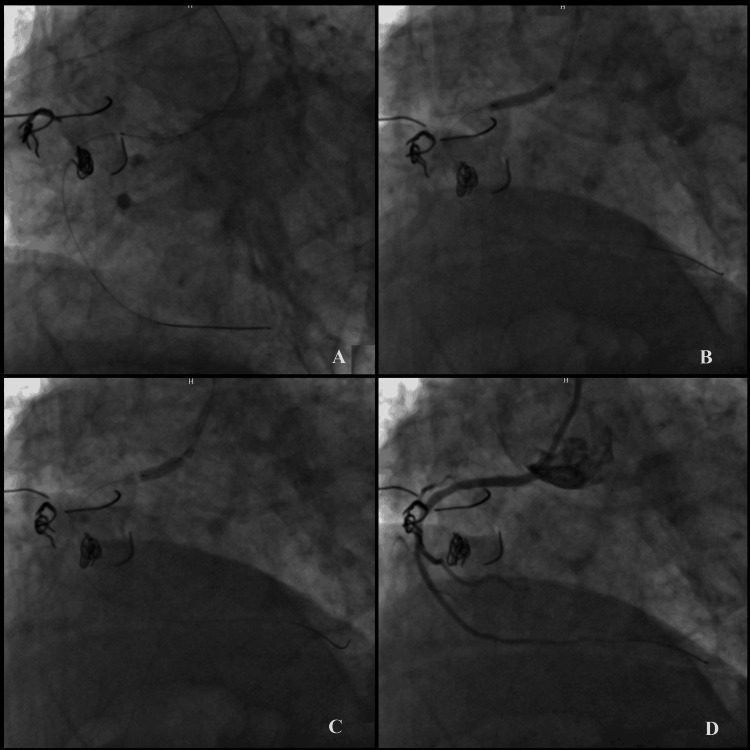
Percutaneous coronary intervention (PCI) to right coronary artery (RCA) ostial lesion (A, B) Sequential balloon dilations and stent implantation; (C) sequential postdilatations; (D) final angiographic image.

It was planned to send a balloon over this more proximal wire to make dilation and create a new ostium for passage. Following sequential balloon dilations performed with 1.0 x 15 mm Sapphire II coronary dilatation catheter (Hong Kong: OrbusNeich), 1.5 x 15 mm Mini Trek Balloon Dilatation Catheter (Abbott) compliant balloons and 2.0 x 15 mm and 2.5 x 15 mm NC Trek Coronary Dilatation Catheter (Abbott) non-compliant balloons, 2.5 x 18 mm Resolute Integrity Zotarolimus-Eluting Stent (Dublin, Ireland: Medtronic) was implanted through the newly formed ostium (Figure 2, panels A and B). Sequential postdilatations were performed with 2.5 x 15 mm and 2.75 x 15 mm NC Trek Coronary Dilatation Catheter (Abbott) non-compliant balloons (Figure 2, panel C). An impressive angiographic result was obtained and PCI was successfully completed without any complications (Figure 2, panel D) The patient had no cardiac complaints at the sixth month of control, and the anginal symptoms were under control.

## Discussion

Intervention of aorto-ostial lesions is a complicated and difficult procedure. Too much overhang of the stent struts into the aorta at the first intervention increases the possibility of in-stent restenosis (ISR), failure of coaxial and selective cannulation of the coronary ostium, and the guidewire not progressing through the true lumen of initial stent. These are major concerns regarding intervention for ostial stent restenosis. These challenges can become more complicated as wiring through the side struts of excessively protruded stent can hamper the advancing of instruments or even provoke stent distortion [5]. After selection of the appropriate guiding catheter, non-selective engagement can be performed by the remote approach to the protruded stent. Excessively overhung stent interventions may require alternative and complex techniques such as double wire technique, balloon-assisted technique, snare technique, side-strut sequential ballooning technique, and guideliner-assisted side-strut stenting technique. Kassimis and Raina published the first reported case of a GuideLiner extension catheter-facilitated side-strut stenting for the treatment of ostial RCA in stent restenosis [3].

Using double wire technique by inserting the first guidewire through the lower side stent strut provides anchor support for the catheter and aligns it more coaxial with the true lumen. As a result, the second guidewire can be advanced distally through the central stent lumen. Chetcuti and Moscuci reported a case of restenosis following ostial stenting of the right coronary artery with protrusion of the stent into the aorta. They used double wire technique in a case in which a 76-year-old female presented with non-ST segment elevation myocardial infarction. Guidewire advanced through a lower strut was used to lever the guide and a second guidewire was advanced through the true lumen [6]. The balloon-assisted method is a refinement of the double wire technique. The first guidewire is advanced distally to stabilize the guiding catheter. Afterward, pushing the balloon catheter towards the ostium of the priorly implanted stent with a slight forward motion enables the retraction of the catheter. Leaving a gap between the overhanging stent ostium and the guiding catheter provides rectification of the target level and the angle in relation to the overhung stent and aids the advancement of the second wire through the central stent lumen [7].

Performing the side-strut sequential balloon technique is relatively simple and easy compared to other methods. The guidewire passes through side-strut of protruding stent and creates a new orifice with sequential ballooning. This technique, however, may result in stent deformation or dislocation with a greater risk of stent embolism [8,9]. Another technique is snare technique which is more complicated than the other techniques. Double arterial access, two guiding catheters, microcatheter, and various guidewires are used for engagement to excessively overhung stent ostium. The microcatheter is advanced via the first guiding catheter and wire through the overhung stent side strut. A j tip-shaped wire through the microcatheter is advanced retrogradely into the aorta and this wire is advanced into the second guiding catheter. Thereafter, the first guiding catheter is pulled out, the second guiding catheter is engaged in the overhung stent ostium, and conventional balloon angioplasty and stent procedures are applied [10].

GuideLiner facilitated side strut stenting is another technique that can be used for excessively overhanging stents to reduce further deformation of struts and to prevent complications related to used angioplasty equipment. One of these techniques can be preferred based on the equipment support and operator experience. On the other hand, several techniques can be used simultaneously as in our case. Here, we used balloon-assisted technique, double wire technique, and side-strut sequential ballooning technique together. It was planned to apply side-strut sequential ballooning method after the first wire had passed through the side strut. However, since the balloon did not pass through the strut, the balloon was left on the first wire and balloon-assisted modified double wire technique was planned. The second wire could pass through the more proximal side strut instead of the central lumen. The side strut was expanded by using sequential balloon dilations and a new ostium was created by passing the guidewire through a more proximal region. As a result, new ostium-mediated stent implantation was performed.

## Conclusions

Intervention of RCA ostial stent restenosis is a challenging angioplasty procedure. During initial stenting, stent size and placement should be very well adjusted to ensure the minimal stent protrusion to the aorta as much as possible. In aorto-ostial stents, following interventions become more complex than the initial stenting, therefore various challenging techniques may be required. Our case emphasizes the necessity of using many methods together in such challenging lesions rather than a single technique.
